# Supplementary material for: Tetraspanin 5 (TSPAN5), a Novel Gatekeeper of the Tumor Suppressor DLC1 and Myocardin-Related Transcription Factors (MRTFs), Controls HCC Growth and Senescence
Source: Cancers (Basel). 2021 Oct 26;13(21):5373. doi: 10.3390/cancers13215373 (PMC8582588; doi:10.3390/cancers13215373)
Supplement: Supplementary file 1 [file cancers-13-05373-s001.zip › cancers-1348628-supplementary.pdf]

Supplementary material for the article:

Tetraspanin 5 (TSPAN5), a novel gatekeeper of the tumor suppressor DLC1 and Myocardin related transcription factor (MRTF), controls HCC growth and senescence

Laura Schreyer[1], Constanze Mittermeier[2], Miriam J. Franz[1], Melanie A. Meier[1], Dietmar E. Martin[3], Kerstin Maier[3], Kerstin Hübner[4], Regine Schneider-Stock[4], Stephan Singer[5], Kerstin Holzer[5], Dagmar Fischer[1], Silvia Ribback[6], Bernhard Liebl[7], Thomas Gudermann[8], Achim Aigner[9] and Susanne Muehlich[1*]

A

Gene Symbol	Fold-Change (HepG2 shRNA DLC1 vs. ctrl)	p-value
VCAN	6.32	0.001515
TSPAN5	4.96	0.001795
MEP1A	4.16	0.002295
HIST1H2BK	3.24	0.003902
CDH2	3.21	0.00091
PODXL	3.10	0.00575
NRP1	2.99	0.041412
PCDHB5	2.64	0.005586
CYP2B6	2.58	0.000419
KLHL13	2.56	0.020755
WDR72	2.54	0.005975
PCDHB16	2.50	0.0117
UIMC1	2.45	0.016427
BCAT1	2.42	0.01225
TNS4	2.35	0.001294
ALDH1L2	2.20	0.002393
GALNT13	2.10	0.022135
MT1A	2.10	0.004688
SESN3	2.09	0.012092
PAR4	-2.03	0.020296
KNG1	-2.03	0.000961
BICC1	-2.05	0.002108
TANC1	-2.09	0.002461
PDGFC	-2.10	0.005088
SLC40A1	-2.11	0.001429
ALB	-2.11	0.00283
SLC44A3	-2.17	0.003617
DTNA	-2.17	0.005333
SNORD115-5	-2.22	0.011644
WDR52	-2.22	0.006268
SHC3	-2.22	0.006272
PAM	-2.23	0.021553
SNORD115-5	-2.35	0.008363
COBL	-2.36	0.011043
SNRPN	-2.39	0.007785
CITED2	-2.41	0.015853
TSPAN7	-2.48	0.002931
TLE4	-2.50	0.040239
UBD	-2.52	0.002018
PSD3	-2.54	0.016774
CHST9	-2.55	0.006465
UBD	-2.56	0.001469
SNORD115-44	-2.63	0.001566
SH3BGRL	-2.65	0.004452
FAM160A1	-2.89	0.012482
HEY1	-2.96	0.00022
UGT2A3	-3.69	0.007205
ALCAM	-13.03	0.001556

B

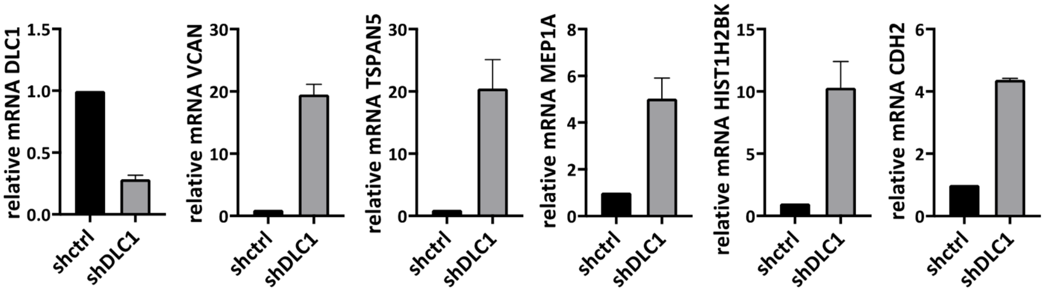

C

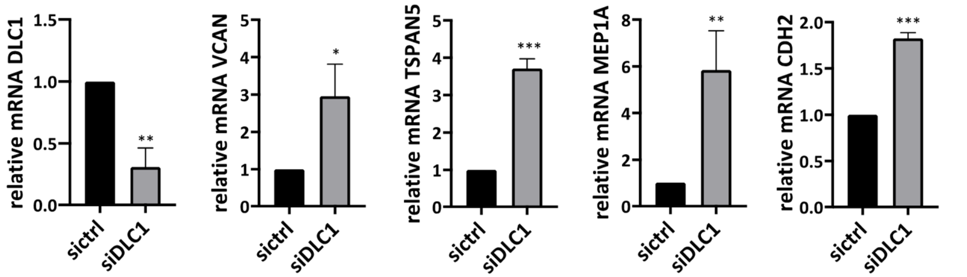

D

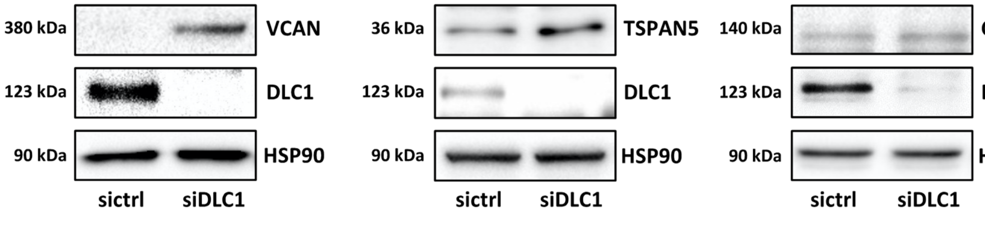

S 1

A

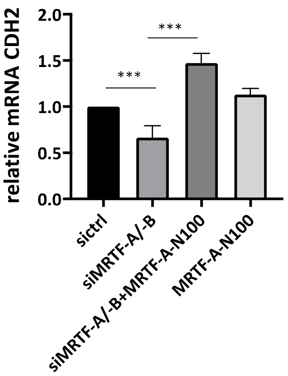
relative mRNA CDH2
2.0
1.5
1.0
0.5
0.0
***
***
sictrl
siMRTF-A/-B
siMRTF-A/-B+MRTF-A-N100
MRTF-A-N100

S 2

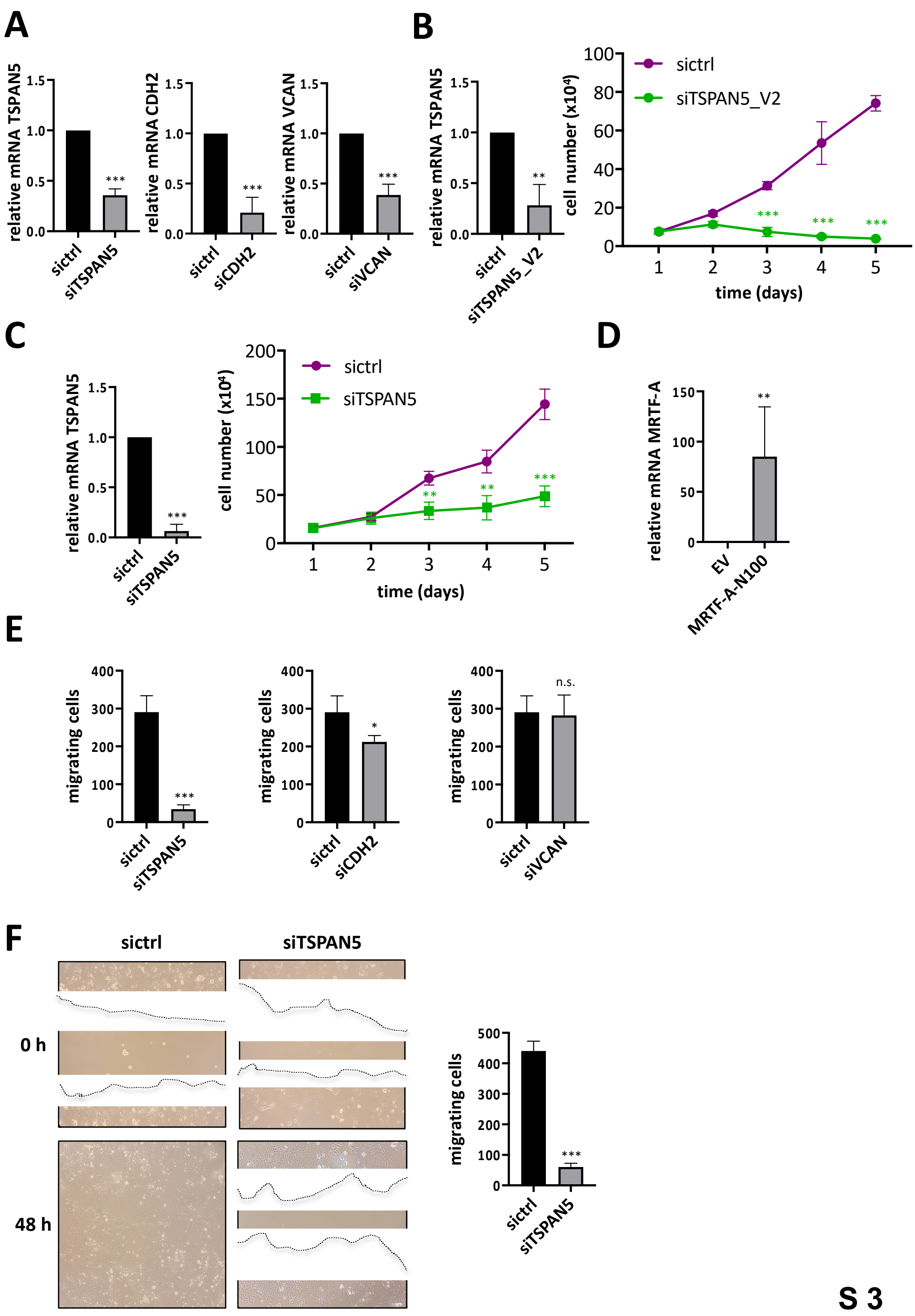

A
relative mRNA TSPAN5
1.5
1.0
0.5
0.0
***
sictrl
siTSPAN5
relative mRNA CDH2
***
siCDH2
relative mRNA VCAN
***
siVCAN
B
**
siTSPAN5_V2
100
80
60
40
20
0
cell number (x10⁴)
sictrl
siTSPAN5_V2
***
1
2
3
4
5
time (days)
C
200
150
100
50
siTSPAN5
**
D
relative mRNA MRTF-A
EV
MRTF-A-N100
E
migrating cells
400
300
200
100
*
n.s.
F
0 h
48 h
500
S 3

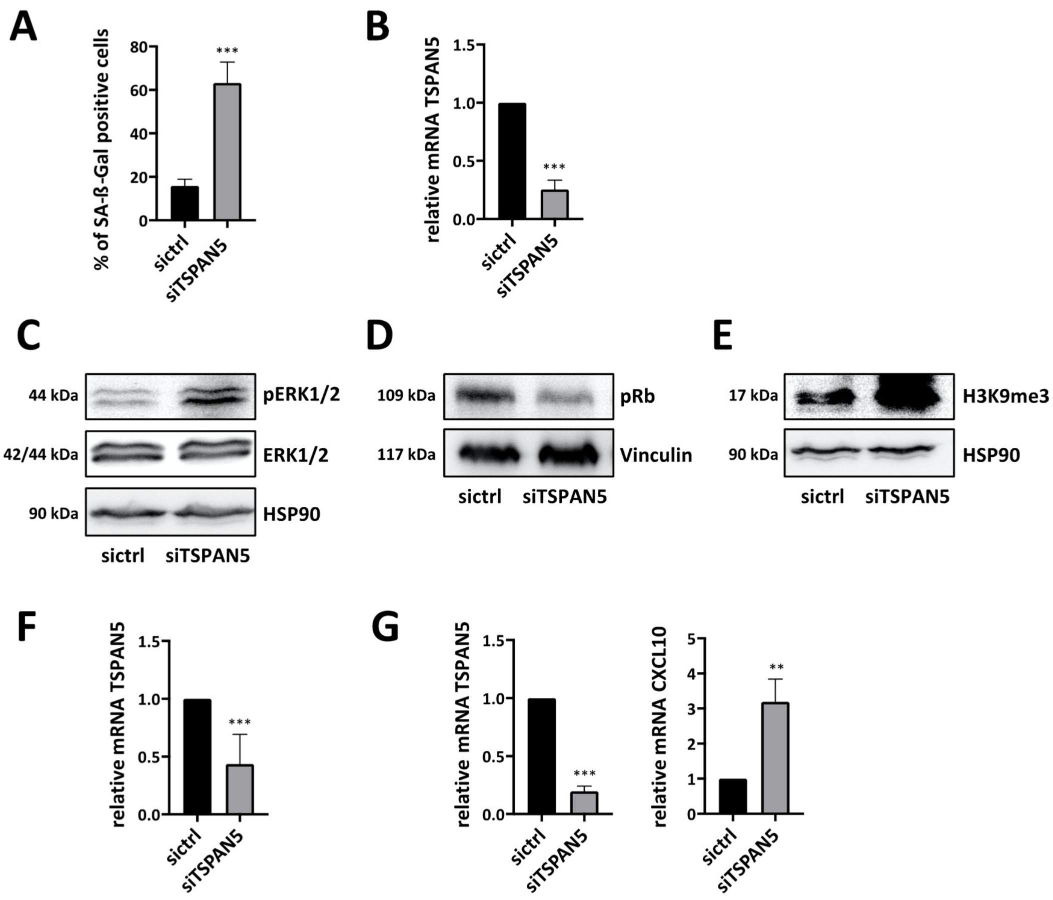
A
% of SA-ß-Gal positive cells
80
60
40
20
0
***
sictrl
siTSPAN5
B
relative mRNA TSPAN5
1.5
1.0
0.5
0.0
***
sictrl
siTSPAN5
C
44 kDa
pERK1/2
42/44 kDa
ERK1/2
90 kDa
HSP90
sictrl
siTSPAN5
D
109 kDa
pRb
117 kDa
Vinculin
sictrl
siTSPAN5
E
17 kDa
H3K9me3
90 kDa
HSP90
sictrl
siTSPAN5
F
relative mRNA TSPAN5
1.5
1.0
0.5
0.0
***
sictrl
siTSPAN5
G
relative mRNA TSPAN5
1.5
1.0
0.5
0.0
***
sictrl
siTSPAN5
relative mRNA CXCL10
5
4
3
2
1
0
**
sictrl
siTSPAN5

S 4

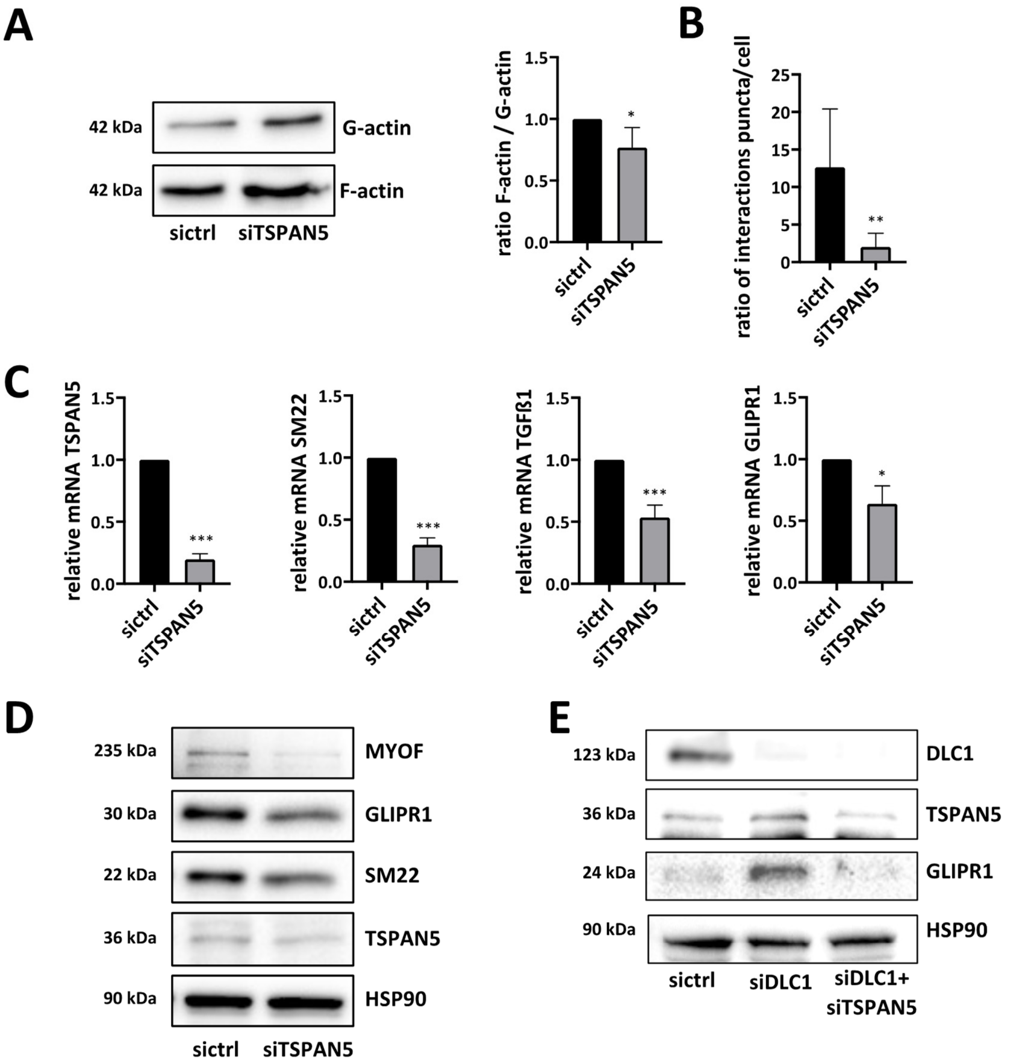
A
42 kDa
G-actin
42 kDa
F-actin
sictrl
siTSPAN5
ratio F-actin / G-actin
1.5
1.0
0.5
0.0
*
B
ratio of interactions puncta/cell
25
20
15
10
5
0
**
C
relative mRNA TSPAN5
relative mRNA SM22
relative mRNA TGFß1
relative mRNA GLIPR1
***
*
D
235 kDa
MYOF
30 kDa
GLIPR1
22 kDa
SM22
36 kDa
TSPAN5
90 kDa
HSP90
E
123 kDa
DLC1
36 kDa
TSPAN5
24 kDa
GLIPR1
90 kDa
HSP90
sictrl
siDLC1
siDLC1+
siTSPAN5

S 5

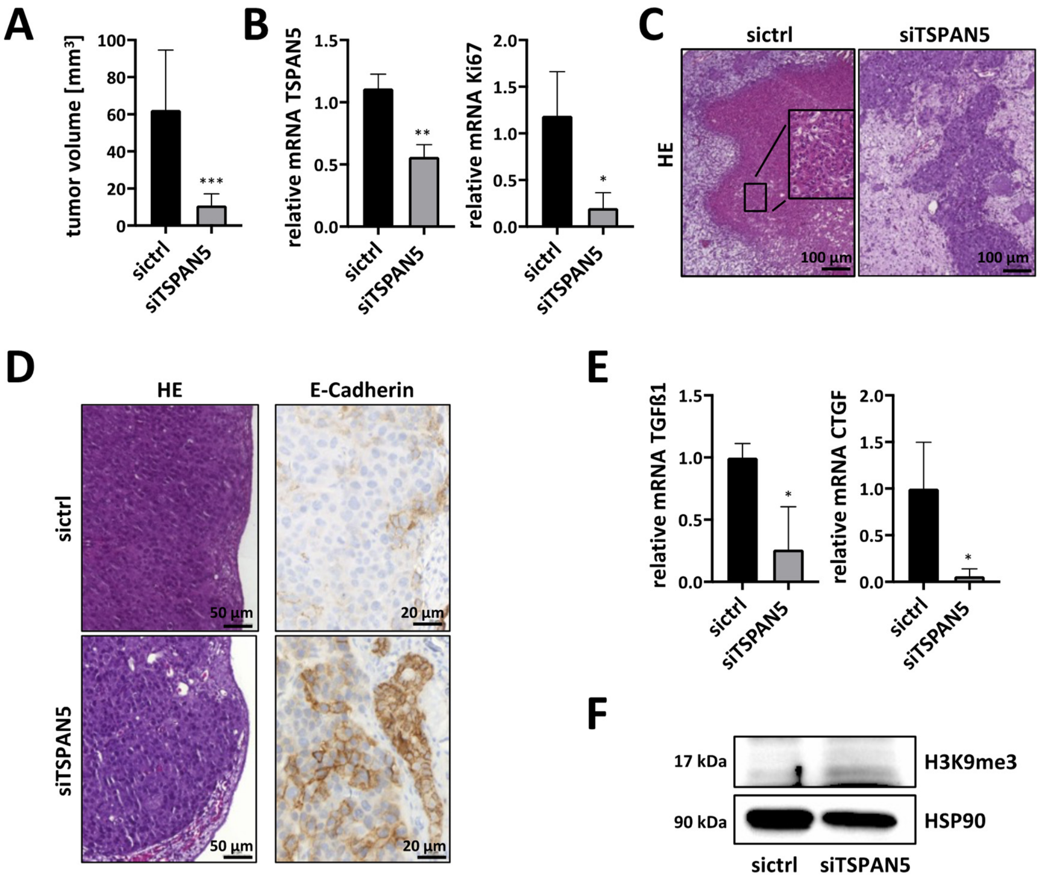
A
tumor volume [mm³]
100
80
60
40
20
0
***
sictrl
siTSPAN5
B
relative mRNA TSPAN5
1.5
1.0
0.5
0.0
**
relative mRNA Ki67
2.0
1.5
1.0
0.5
0.0
*
C
sictrl
siTSPAN5
HE
100 µm
D
HE
E-Cadherin
50 µm
20 µm
E
relative mRNA TGFß1
relative mRNA CTGF
F
17 kDa
H3K9me3
90 kDa
HSP90

A

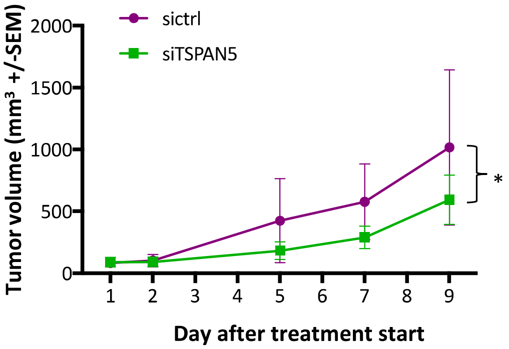

sictrl
siTSPAN5
Tumor volume (mm³ +/-SEM)
2000
1500
1000
500
0
1
2
3
4
5
6
7
8
9
Day after treatment start
*

Fig. S1: Complete results and validation of gene expression profiling

A) DNA microarray analysis was performed to analyse the transcriptome of HepG2 cells either expressing control shRNA (ctrl) or DLC1 shRNA (DLC1 KD). Genes are ordered to the average fold of downregulation upon DLC1 depletion and the corresponding p-values are given.

B) Knockdown efficiency of the stable DLC1 shRNA knockdown and expression levels of DLC1 gene targets in HepG2 ctrl and HepG2 DLC1KD cells, determined by qRT-PCR using gene-specific primers and normalized to the endogenous housekeeping gene 18 S rRNA. Data are means ± SD (n=2).

C) Expression levels of the indicated target genes in Hep3B cells expressing sictrl or siDLC1 cells and knockdown efficiency of DLC1, measured by qRT-PCR. Values are mean ± SD (n=3); *p<0.05, **p<0.01, ***p<0.001.

D) Lysates of Hep3B cells expressing ctrl siRNA and DLC1siRNA were subjected to immunoblotting with anti-Versican, anti-n-Cadherin, anti-TSPAN5 or anti-HSP90 antibody as loading control. Representative blots of three independent experiments are shown.

Fig. S2: Transcriptional regulation of CDH2 by MRTF-A/B

A) Expression of CDH2 mRNA in HuH7 cells after RNAi-mediated MRTF-A/-B knockdown (siMRTF-A/-B), followed by ectopic expression of MRTF-A-N100 for 3 days, determined by qRT-PCR. The respective gene specific primers were used and normalization to the 18 S rRNA was carried out. Values are mean ± SD (n=3); ***p<0.001.

Fig. S3: Requirement of TSPAN5 for HCC proliferation and migration

A) Knockdown efficiencies in HuH7 cells transfected with negative control (sictr) and TSPAN5, CDH2 or VCAN siRNAs were determined by qRT-PCR after 48 h using the appropriate primers listed in table 7 and 18S rRNA primers for normalization. Values are mean ± SD (n=3); ***p<0.001.

B) HuH7 cells transfected with negative control and an alternative version of TSPAN5 siRNA V2 (siTSPAN5_V2) were counted daily for 5 days (right). Knockdown efficiency of mRNA expression was determined by qRT-PCR after 48 h using TSPAN5 and 18S rRNA primers for normalization. Values are mean ± SD (n=3); **p<0.01, ***p<0.001.

C) HuH6 cells transfected with TSPAN5 siRNA (siTSPAN5) and scrambled RNA (sictrl) were assessed by qRT-PCR as above and by counting with a Neubauer chamber for 5 days. Values are mean ± SD (n=3); **p<0.01, ***p<0.001.

D) HuH7 cells expressing TSPAN5 siRNA were transfected with FLAG-tagged empty vector (EV) and MRTF-A-N100 for 48 h were subjected to qRT-PCR as above. Values are mean ± SD (n=3); **p<0.01.

E) Cell migration of HuH7 cells transfected with the indicated siRNAs, assessed by a culture scratch-wound assay and determining the migrated cells per part of scratch. Values are mean ± SD (n=3); *p<0.05, ***p<0.001.

F) Cell migration of HuH6 cells expressing scrambled siRNA (sictrl) or TSPAN5 siRNA (siTSPAN5) determined by scratch-wound assay as above. Values are mean ± SD (n=3); ***p<0.001.

Fig. S4: Requirement of TSPAN5 for oncogene-induced senescence in HCC

A) Quantification of senescence associated ß-galactosidase positive cells in HuH6 cells transiently transfected with control siRNA (sictrl) in comparison to TSPAN5 siRNA (siTSPAN5). ß-galactosidase staining was performed 5 days after transfection and ß-gal positive cells were counted in 100 cells per condition. Data are means ± SD (n=3); ***p<0.001.

B) Knockdown efficiency of TSPAN5 in HuH7 cells transfected with control siRNA (sictrl) and with TSPAN5 siRNA (siTSPAN5) determined by qRT-PCR with TSPAN5 and 18S rRNA primers for normalization. Data are means ± SD (n=3); ***p<0.001.

(C-E) Lysates of HuH6 cells transiently transfected with negative control siRNA (sictrl) or TSPAN5 siRNA (siTSPAN5) for 6 days were immunoblotted with anti-pERK1/2, anti-ERK and anti-HSP90 **(C)**, anti-pRB and anti-HSP90 **(D)** and anti-H3K9me3 and anti-HSP90 antibodies **(E)**. Representative blots of three independent experiments are shown.

F) HuH7 cells transfected with negative control (sictrl) or TSPAN5 siRNA (siTSPAN5) were subjected to qRT-PCR with TSPAN5 primers and 18S rRNA primers for normalization to analyze TSPAN5 kockdown efficiency with refer to experiment, which is shown in Fig. 4G. Values are mean ± SD (n=3); ***p<0.001.

G) *TSPAN5* and *CXCL10* mRNA expression in HuH6 cells transfected with negative control (sictrl) or TSPAN5 siRNA (siTSPAN5), determined by qRT-PCR after 6 days with the respective gene specific primers and 18S rRNA primers for normalization. Values are mean ± SD (n=3); **p<0.01, ***p<0.001.

Fig. S5: Depletion of TSPAN5 affects the actin/MRTF signaling axis in HCC

A) G- and F-actin fractionation in HuH6 cells transiently transfected with TSPAN5 siRNA (siTSPAN5) compared to negative control siRNA (sictrl). Data are means ± SD (n=3); *p< 0.05.

B) Quantification of immunofluorescence analysis of proximity ligation assay (PLA) for endogenous MRTF-A and FLNA in 3T3 cells transiently transfected with TSPAN5 siRNA (siTSPAN5) compared to negative control siRNA (sictrl). Scale bar, 10 µm. PLA signals were counted in 15 cells per condition. All data are means ± SD (n=3); **p< 0.01.

C) Expression levels of the MRTF target genes SM22, TGFß1 and GLIPR1 and TSPAN5 in HuH6 cells transiently transfected with TSPAN5 siRNA (siTSPAN5) versus scrambled RNA (sictrl), determined by qRT-PCR with normalization to 18S rRNA with specific primers for SM22, TGFß1, GLIPR1 and TSPAN5. Values are mean ± SD (n=3); *p<0.05, ***p<0.001.

D) Lysates of HuH7 cells transiently transfected with negative control siRNA (sictrl) or TSPAN5 siRNA (siTSPAN5) for 3 days were immunoblotted with anti-Myoferlin (MYOF), anti-GLIPR1, anti-Transgelin (SM22), anti-Tetraspanin 5 (TSPAN5) and anti-HSP90 antibody as loading control. Representative blots of three independent experiments are shown.

E) Lysates of HepG2 cells expressing negative control siRNA (sictrl), DLC1 siRNA (siDLC1) or DLC1 siRNA combined with TSPAN5 siRNA (siDLC1 + siTSPAN5) were immunoblotted with anti-DLC1, anti-Tetraspanin 5, anti-GLIPR1 and anti-HSP90 antibody as loading control. Representative blots of three independent experiments are shown.

Fig. S6: Anti-tumor effects of TSPAN5 depletion in the chorio-allantoic membrane (CAM) assay

A) HepG2 clone 5 cells transfected with negative control siRNA (sictrl) and TSPAN5 siRNA (siTSPAN5) in Matrigel® pellets were transferred to the CAM of fertilized chicken eggs. 5 days later CAM tumor pellets of HepG2 clone 5 cells were extracted, ex ovo images of micro-tumors were taken and tumor volume was calculated. Data are presented with means ± SD (n=15); ***p<0.001.

B) Knockdown efficiency of TSPAN5 and Ki67 expression in micro-tumors generated in A), determined by qRT-PCR using TSPAN5, Ki67 and 18S primers as endogenous housekeeping gene for normalization. Values are mean ± SD (n=3); *p<0.05, **p<0.01.

C) Representative photomicrographs of hematoxylin-eosin (HE) stained paraffin sections of HuH7 sictrl and siTSPAN5 CAM tumors. Scale bar = 100 µm.

D) Left panel: Representative images of hematoxylin-eosin (HE) and TSPAN5 stained paraffin sections of HepG2 clone 5 sictrl and siTSPAN5 CAM tumors generated as in A). Scale bar = 50 µm. Right panel: Photomicrographs of TSPAN5 and E-Cadherin stained paraffin sections of HepG2 clone 5 sictrl and siTSPAN5 CAM tumors prepared as in A). Scale bar = 20 µm.

E) RNA was purified from micro tumors derived from HuH7 cells treated with scrambled siRNA (sictrl) or TSPAN5 siRNA (siTSPAN5) for 5 days in ovo and TGFß1 and CTGF expression assessed by qRT-PCR as in B). Values are mean ± SD (n=3); *p<0.05.

F) Lysates from extracted CAM tumors derived from HepG2 cells transfected with scrambled siRNA (sictrl) and TSPAN5 siRNA (siTSPAN5) were immunoblotted with anti-H3K9me3 antibody and anti-HSP90 antibody as a loading control. Representative blots of three independent experiments are shown.

Fig. S7: Anti-tumor effects of TSPAN5 depletion in the mouse xenograft

A) HuH7 HCC xenograft bearing mice were randomized and treated on day 2, 4, 6 and 8 by systemic injection with polymeric nanoscale complexes containing siTSPAN5 or sictrl. Tumor growth was measured manually. Means of tumor sizes are shown for control and treated group. Data are presented with means ± SD (n=6 mice per group).

Table S1: Cell lines and their culture medium

Cell line	Culture medium	Manufacturer (medium)
**3T3**	DMEM (Dulbecco's modified Eagle's medium)	Merck, Darmstadt, Germany
**A7**	MEM (Minimum Essential Medium Eagle)	Merck, Darmstadt, Germany
**Hep3B**	DMEM (Dulbecco's modified Eagle's medium)	Merck, Darmstadt, Germany
**HepG2**	RPMI 1640 (Roswell Park Memorial Institute medium)	Merck, Darmstadt, Germany
**HepG2 DLC1 CRISP Cas9 KO**	RPMI 1640 (Roswell Park Memorial Institute medium)	Synthego Corporation, Menlo Park, CA, USA
**HepG2 DLC1 CRISP Cas9 wt**	RPMI 1640 (Roswell Park Memorial Institute medium)	Synthego Corporation, Menlo Park, CA, USA
**HuH6**	DMEM (Dulbecco's modified Eagle's medium)	Merck, Darmstadt, Germany
**HuH7**	RPMI 1640 (Roswell Park Memorial Institute medium)	Merck, Darmstadt, Germany
**M2**	MEM (Minimum Essential Medium Eagle)	Merck, Darmstadt, Germany

Table S2: siRNA sequences used for transient knockdown

siRNA	Sequence
**siRNA control**	5′-CGUACGCGGAAUACUUCGA[dT][dT]-3′
**siCDH2**	5′-AAAGUGGCAAGUGGCAGUAAA[dT][dT]-3′
**siDLC1**	5′-UUAAGAACCUGGAGGACUA[dT][dT]-3′
**siFLNA**	5′-GCACAUGUUCCGUGUCCUA[dT][dT]-3′
**siMRTF-A/-B**	5′-AUGGAGCUGGUGGAGAAGAA[dT][dT]-3′
**siTSPAN5**	5′-GAGCAUAUCGGGAUGACAU[dT][dT]-3′
**siTSPAN5 V2**	5′-GACCAGCUGUAUUUCUUUA[dT][dT]-3′
**siVCAN**	5′-GAGGCUGGAACUGUUAUUA[dT][dT]-3′

Table S3: Plasmid constructs used for transient transfection

Plasmid construct	Manufacturer
**pEFrPuro-Flag-DLC1**	Kind gift of Prof. Monilola Olayioye, University of Stuttgart, Stuttgart, Germany
**p3xFlag MRTF-A-N100**	Kind gift of Prof. Ron Prywes, Columbia University, NY, USA
**p3xFlag 7.1**	Merck, Darmstadt, Germany

**pCMV6-AC-GFP TSPAN5**	OriGene Technologies, Inc., Rockville, MD, USA
**pEGFP-N1**	Merck, Darmstadt, Germany

Table S4: Primer sequences used for qRT-PCR

qRT-PCR primer	sequence
**18S Fw**	5′-TCG AGG CCC TGT AAT TGG AAT-3′
**18S Rv**	5′-CCC TCC AAT GGA TCC TCG TTA-3′
**CDH2 Fw**	5′-CTC CAT GTG CCG GAT AGC-3′
**CDH2 Rv**	5′-CGA TTT CAC CAG AAG CCT CTA C-3′
**CNN1 Fw**	5′-GCT GTC AGC CGA GGT TAA GA-3′
**CNN1 Rv**	5′-CCC TCG ATC CAC TCT CTC AG-3′
**CTGF Fw**	5′-TTG GCA GGC TGA TTT CTA GG-3′
**CTGF Rv**	5′-GGT GCA AAC ATG TAA CTT TTG G-3′
**CXCL10 Fw**	5′-CCC CAC GTT TTC TGA GAC AT-3′
**CXCL10 Rv**	5′-TGG CAG TTT GAT TCA TGG TG-3′
**DLC1 Fw**	5′-GAG CAG TGT CAT GCC TTG G-3′
**DLC1 Rv**	5′-GCG AAT GAG TTC TGT CAT TTC A-3′
**GLIPR1 Fw**	5′-TCT TTC CAA TGG AGC ACA TTT-3′
**GLIPR1 Rv**	5′-TCT TAT ATG GCC AAG TTG GGT AA-3′
**HIST1H2KB Fw**	5´-ACC TCC AGG GAG ATC CAG AC -3´
**HIST1H2KB Rv**	5´-TCC AGA GAA AGT CCC TCC TGG -3
**Ki67 Fw**	5´-TCA AGG ACC TGA TTC AGG AGA AG -3´
**Ki67 Rv**	5´-GTG CAC TGA AGA ACA CAT TCC -3´
**Mep1A Fw**	5′-CTT GTT GGG ACA ATG CAC AG-3′
**Mep1A Rv**	5′-GGG TAA AGA ATC CGA GAC TCC-3′
**MRTF-A Fw**	5′-CCC AAT TTG CCT CCA CTT AG-3′
**MRTF-A Rv**	5′-CCT TGG CTC ACC AGT TCT TC-3′
**SMA Fw**	5′-CCT ATC CCC GGG ACT AAG AC-3′
**SMA Rv**	5′-AGG CAG TGC TGT CCT CTT CT-3′
**SM22 Fw**	5′-GGC CAA GGC TCT ACT GTC TG-3′
**SM22 Rv**	5′-CCC TTG TTG GCC ATG TCT-3′
**SRF Fw**	5′-AGC ACA GAC CTC ACG CAG A-3′
**SRF Rv**	5′-GTT GTG GGC ACG GAT GAC-3′
**TGFβ1 Fw**	5′-ACT ACT ACG CCA AGG AGG TCA C-3′
**TGFβ1 Rv**	5′-TGC TTG AAC TTG TCA TAG ATT TCG-3′
**TNFSF10 Fw**	5′-TTC ACA GTG CTC CTG CAG TC-3′
**TNFSF10 Rv**	5′-GCC ACT TTT GGA GTA CTT GTC C-3′
**TSPAN5 Fw**	5′-ATG CAA GTC GAG AGC GAT GT-3′
**TSPAN5 Rv**	5′-GGC ATC ATA GCC ACA CTG AG-3′
**VCAN Fw**	5′-GCA CCT GTG TGC CAG GAT A-3′
**VCAN Rv**	5′-CAG GGA TTA GAG TGA CAT TCA TCA-3′
**Random Hexamers**	5′-NNN NNN-Wobbles-3′

Table S5: Kits used for microarray analysis

Kit	Manufacturer
**RNeasy Mini Kit**	Quiagen, Hilden, Germany
**Ambion WT Expression Kit**	Life Technologies, Darmstadt, Germany
**GeneChip WT Terminal Labeling Kit**	Affymetrix, Santa Clara, CA, USA

Table S6: Antibody used for chromatin immunoprecipitation (ChIP)

Antibody	Manufacturer
**anti-MRTF-A**	Santa Cruz Biotechnology, Santa Cruz, CA, USA
**anti-IgG**	Cell Signaling Technologies, Danvers, MA, USA

Table S7: Primer sequences used for ChIP qRT-PCR

ChIP qRT-PCR primer	sequence
**CDH2 Fw**	5′-ACCCAGAGATCAAGGAGGTG-3′
**CDH2 Rv**	5′-CTCCACTTCCACCTCCACAT-3′
**TSPAN5 Fw**	5′-GCTCATCAATCCCGGTCA-3′
**TSPAN5 Rv**	5′-GGCGAGAGGGAGAAGGAA-3′
**VCAN Fw**	5′-ACCTCTTGGCGTTTCTTCCT-3′
**VCAN Rv**	5′-CTCCTTTCCCCTAACCCAGA-3′

Table S8: Primary antibodies used for immunoblotting

Antibody	Manufacturer
**anti-Actin (rabbit)**	Merck, Darmstadt, Germany
**anti-DLC1 (mouse)**	BD Bioscience, San Jose, CA, USA
**anti-Erk1/2 (p44/42 MAPK) (rabbit)**	Cell Signaling Technology, Danvers, MA, USA
**anti-GLIPR1 (rabbit)**	Santa Cruz Biotechnology, Inc., Dallas, TX, USA
**anti-H3K9me3 (rabbit)**	Actif Motif, Carlsbad, USA
**anti-HSP90 (mouse)**	Santa Cruz Biotechnology, Inc., Dallas, TX, USA
**anti-Myoferlin (mouse)**	Santa Cruz Biotechnology, Inc., Dallas, TX, USA
**anti-N-Cadherin (rabbit)**	Cell Signaling Technology, Danvers, MA, USA
**anti-p16^{INK4a} (goat)**	R&D Systems, Inc., Minneapolis, MN, USA
**anti-phospho-p44/42 MAPK (Erk1/2) (Thr202/Tyr204) (rabbit)**	Cell Signaling Technology, Danvers, MA, USA

**anti-pRb (mouse)**	BD Bioscience, San Jose, CA, USA
**anti-active RhoA**	NewEast Bioscience, Malvern, PA, USA
**anti-RhoA polyclonal antibody**	NewEast Bioscience, Malvern, PA, USA
**anti-SMA (rabbit)**	Abcam, Cambridge, UK
**anti-SRF (rabbit)**	Santa Cruz Biotechnology, Inc., Dallas, TX, USA
**anti-Transgelin (SM22) (mouse)**	Merck, Darmstadt, Germany
**anti-TSPAN5 (rabbit)**	Merck, Darmstadt, Germany
**anti-Versican (mouse)**	Santa Cruz Biotechnology, Inc., Dallas, TX, USA
**anti-Vincullin (mouse)**	Merck, Darmstadt, Germany

Table S9: Primary antibodies used for proximity ligation assay

**Primary antibody**	**Manufacturer**
**anti-MRTF-A**	Santa Cruz Biotechnology, Santa Cruz, CA, USA
**anti-FLNA**	Millipore, Merck, Darmstadt, Germany

Table S10: Primary antibodies used for immunohistochemistry

**Antibody**	**Dilution**	**Manufacturer**
**anti-E-Cadherin, polyclonal (mouse)**	1:2000	BD Bioscience, San Jose, CA, USA
**anti-H3K9me3, polyclonal (rabbit)**	1:200	Merck, Darmstadt, Germany
**anti-Ki-67, monoclonal (mouse)**	1:100	Dako/Agilent, Santa Clara, CA, USA
**anti-p16, monoclonal (mouse)**	1:20	BD Bioscience, San Jose, CA, USA
**Anti-rabbit, biotinylated (goat)**	1:100	Vector-Laboratories, Burlingame, CA, USA
**anti-TSPAN5, polyclonal (rabbit)**	1:200	OriGene Technologies, Rockville, MD, USA

Tabelle S11: Patient characteristics including age, gender, grading, staging, lymphangiosis and hemangiosis carcinomatosa, R-status and tumor size

**PATIENT CHARACTERISTICS**		**HCC**
**Age (years)**	Mean ± SD	69.8 ± 7.5
	Range	61 - 89
**Gender**	F	-
	M	11

**Grading**	G1	5
	G2	6
**Stage (7th edition UICC)**	T1	6
	T2	3
	T3	1
	unknown	1
**Lymphangiosis**	yes	-
	no	9
	unknown	2
**Hemangiosis**	yes	3
	no	7
	unknown	1
**R-Status**	R0	10
	R1	-
	unknown	1
**Tumour size cm (n=10)**	Mean ± SD	5 ± 5
	Range	2 – 19
